# 2nd-Order Debye relaxation in electromagnetic metasurfaces for wideband dispersion engineering

**DOI:** 10.1038/s41377-025-01813-1

**Published:** 2025-03-27

**Authors:** Xinmin Fu, Yajuan Han, Jiafu Wang, Jie Yang, Yong Sun, Chang Ding, Yuxiang Jia, Jun Wang, Shaobo Qu, Tiejun Cui

**Affiliations:** 1 Shaanxi Key Laboratory of Artificially-Structured Functional Materials and Devices, Air Force Engineering University, 710051 Xi’an, China; 2 Suzhou Laboratory, 215000 Suzhou, Jiangsu China; 3 Institute of Electromagnetic Space, Southeast University, 210096 Nanjing, China

**Keywords:** Metamaterials, Sub-wavelength optics

## Abstract

In dielectric physics, electromagnetic (EM) properties of dielectrics arise from several important polarization mechanisms that can be described by Debye, Drude or Lorentz models. Metamaterials, as well as their 2D counterparts-metasurfaces, can exhibit bizarre EM parameters such as negative permittivity, whereas polarization mechanisms leading to such have long been discussed in dielectric physics. Drude and Lorentz's models are usually used in metamaterial design, whereas the Debye model is almost absent, though it is so important in dielectric physics. This leaves an unreconciled gap between the dielectric physics and metamaterials. In this paper, we explore Debye relaxations in metasurfaces for the sake of wideband dispersion engineering. By analyzing two fundamental resonance modes of a typical meta-atom, we first show that the reflection phase experiences 1^st^-order Debye relaxation under the two resonances, although they are typically Lorentzian. More importantly, the two resonances can be tailored to form a 2nd-order Debye relaxation process so as to achieve smooth phase variations in between them, which lays a solid foundation for wideband dispersion engineering. As proof of concept, we propose a quad-elliptical-arc (QEA) structure as the meta-atom, whose dispersion can be customized by tailoring the 2nd-order Debye relaxation. With this meta-atom, we demonstrated two metasurface prototypes that can achieve chromatic and achromatic focusing, respectively, in the entire X band (8.0–12.0 GHz), showcasing the powerful capacity of wideband dispersion engineering. This work digs out relaxation processes in metamaterials and opens up new territories for metamaterial research, which may find wide applications in wideband devices and systems.

## Abstract (web summary)

A second-order Debye relaxation process has been demonstrated in electromagnetic metasurfaces, offering enhanced degrees of freedom for broadband dispersion engineering.

## Graphical abstract

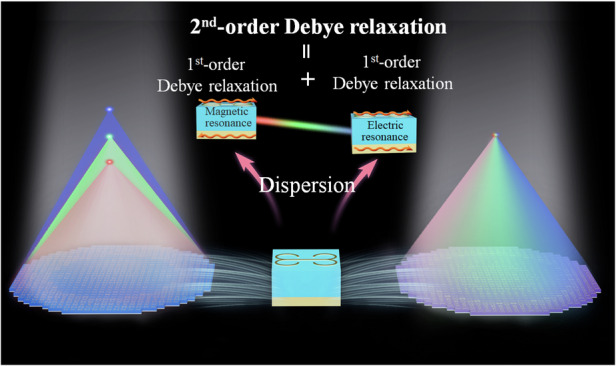

## Introduction

Polarization is one of the most important EM properties of dielectric materials. The essence of polarization is electron movement, manifested as displacements or rotation of electrons with respect to the centers of molecules/atoms in dielectrics or collective oscillation of free electrons in metals, which produces aligned dipole moments along the direction of external electric fields. Polarization is very fundamental in revealing the microscopic mechanisms of macroscopic EM properties of dielectrics^1–3^. For conventional dielectrics, their constituent particles include atoms, molecules, and others^4–6^. In general, without external electric fields, the contribution of these particles to macroscopic polarization vanishes due to thermal motion averaging. It is only under the influence of applied electric fields that particles align along the field direction and contribute to dipole moments that can give rise to macroscopic polarization intensity.

**Fig. 1:**
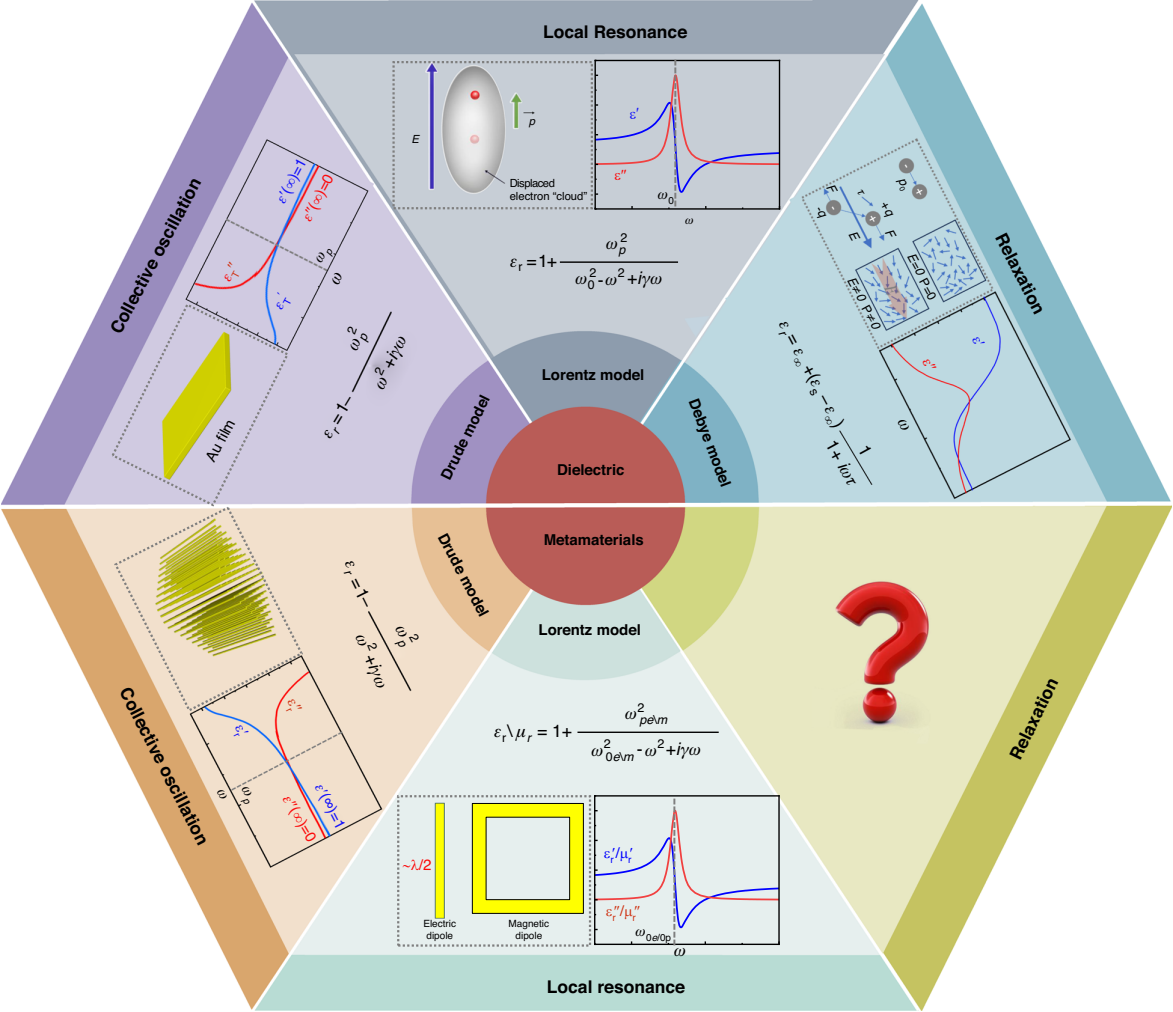
The dielectric response models for conventional dielectric materials and for metamaterials. For conventional dielectric materials, there are three types of polarization models: the Drude model for collective oscillation of free electrons, the Lorentz model for local resonance of electric dipoles, and the Debye model for the relaxation process of dipole orientation. In contrast, for metamaterials, there are only two models: the Drude model for infinitely long wires and the Lorentz model for resonant structures (e.g., short metallic wires and split-ring resonators (SRRs)). The relaxation model is absent in metamaterial research

For a single molecule/atom, the relationship between the induced electric dipole moment and external electric fields can be expressed as **P** = *α***E**, where *α* is the electric polarizability. The generation of polarizability mainly originates from three mechanisms, namely, dipole orientation polarization *α*_d_, atom (or ion) displacement polarization *α*_i_, and electron displacement polarization *α*_e_. The macroscopic EM properties of dielectrics can be ascribed to these induced dipole moments, which are usually averaged by volume and normalized by the permittivity of vacuum, that is, the electric susceptibility. The concept of electric susceptibility describes the influences of dielectrics on EM waves macroscopically and it is in nature a volume-averaged and vacuum-normalized concept. Plus the relative permittivity of vacuum, we then obtain the commonly known concept of the relative permittivity (or the so-called dielectric constant in material science), which is just the macroscopic manifestation of polarizability in dielectrics. Due to the existence of dielectrics, there is a noticeable phase difference between electric displacement **D** and electric field **E**, which can be described by relative permittivity *ε*_r_ = *ε*′_r_ + *jε*″_r_ from the perspective of constitutive parameters. According to the spectra characteristics, the variation of relative permittivity with frequency can be described by three models: The drude model for collective oscillation of free electrons within metals, the Lorentz model for localized resonance of confined/bound electrons, and the Debye model for relaxation process of dipole orientation, as illustrated in Fig. 1. Both the electron displacement polarization and atom displacement polarization can be described by Lorentz model, and they can follow the variations of external fields swiftly, thereby exhibiting resonance characteristics with short relaxation times. In contrast, the establishment time of dipole orientation polarization is relatively long, rendering it unable to fully respond to changes in external electric fields. Therefore, polarization relaxation is predominantly caused by dipole orientations.

Metamaterials are artificial composite materials comprising of 2D or 3D arrays of sub-wavelength metallic or dielectric structures (called meta-atoms), which can exhibit bizarre EM parameters such as negative permittivity, near-zero permittivity or extremely large permittivity^7,8^. Since meta-atoms are usually smaller than 1/10 wavelength, the macroscopic EM parameters (permittivity, permeability, refractive index, and others) can be described by the effective medium theory, and thus, metamaterials can be treated as a kind of generalized dielectric material. In 1996, Pendry derived the principles of negative permittivity in microwave regimes by exploiting the Drude model and experimentally verified this idea using thin metallic wires^9,10^. Subsequently, negative permeability was also verified based on the Lorentz model of SRRs. In 2004, Smith et al. constructed the first double-negative metamaterial by combining Drude and Lorentz models^11^. Henceforth, Drude and Lorentz's models have become the most important theoretical models for metamaterials, based on which many metamaterials have been designed and implemented. Due to the high degree of freedom of design, metamaterials can be readily used to manipulate many properties of EM waves, such as amplitude^12^, phase^13,14^, polarization^15–17^, dispersion^18,19^, thus giving birth to various exciting applications such as retroreflection^20,21^, anomalous reflection^22–24^, holographic imaging^25,26^, achromatic focusing^27–29^, and vortexs^30,31^. The bizarre EM properties of metamaterials also arise from the polarization of meta-atoms. From this perspective, metamaterials can be considered as a derivative branch developed from dielectric physics^32,33^. However, until now, almost all metamaterials are interpreted using the Drude and Lorentz models, while the Debye model is almost absent in metamaterial research though it is so important in dielectric physics, as is depicted in Fig. 1. This leaves a flawed gap between the dielectric physics and metamaterials. In fact, Debye relaxation, if exists, is crucial for realizing wideband dispersion engineering of metamaterials since the relaxation process usually spans wide in frequency spectra. Therefore, it would be of great significance if Debye relaxation could be explored in metamaterials and metasurfaces so that researchers and engineers can tailor the dispersion in a wide band to facilitate wideband applications such as radar systems, holographic imaging, new-generation communication, and others.

In this work, we explore relaxation processes in metamaterials and propose to achieve higher-order Debye relaxation by tailoring two adjacent resonances in the EM response spectra of metasurfaces. Since relaxation usually occurs during the transition process from one state to another state, there must be at least two distinct states for the EM response. To render a smooth transition between the two states, the resonance frequency, resonance strength, quality factors, and other features of the two states must be tailorable with a high degree of freedom, which is just the very merit of metamaterials. To this end, we first analyze the two fundamental resonance modes (electric and magnetic resonances) of one of the most typical meta-atom structures (the short-wire structure), which can alter the effective refractive index and thus change the reflection phase versus frequency. We show that the reflection phase experiences 1st-order Debye relaxation under the two resonances, although they are typically Lorentzian. More importantly, the two resonances can be tailored to form a 2nd-order Debye relaxation process. Theoretical model indicates that the effective refractive index can be changed gradually and smoothly between the two resonances. This is quite exciting since it provides a new degree of freedom for customized wideband dispersion engineering. As proof of concept, we propose a quad-elliptical arc (QEA) structure as the meta-atom for wideband metasurface design, which can exhibit 2nd-order Debye relaxation behavior. Based on dispersion engineering enabled by 2nd-order Debye relaxation, we successfully implemented two wideband metasurface prototypes with chromatic and achromatic focusing, respectively. Both simulated and measured results verify the design and thus verify the powerful capability of wideband dispersion engineering via the 2nd-order Debye relaxation. This work reveals 1st- and higher-order relaxation behavior in metamaterials and fills the gap between dielectric physics and metamaterials, which will open up new territories for metamaterial research and may find wide applications in wideband devices and systems.

## Results

### 1st-Debye relaxations in metasurfaces

**Fig. 2:**
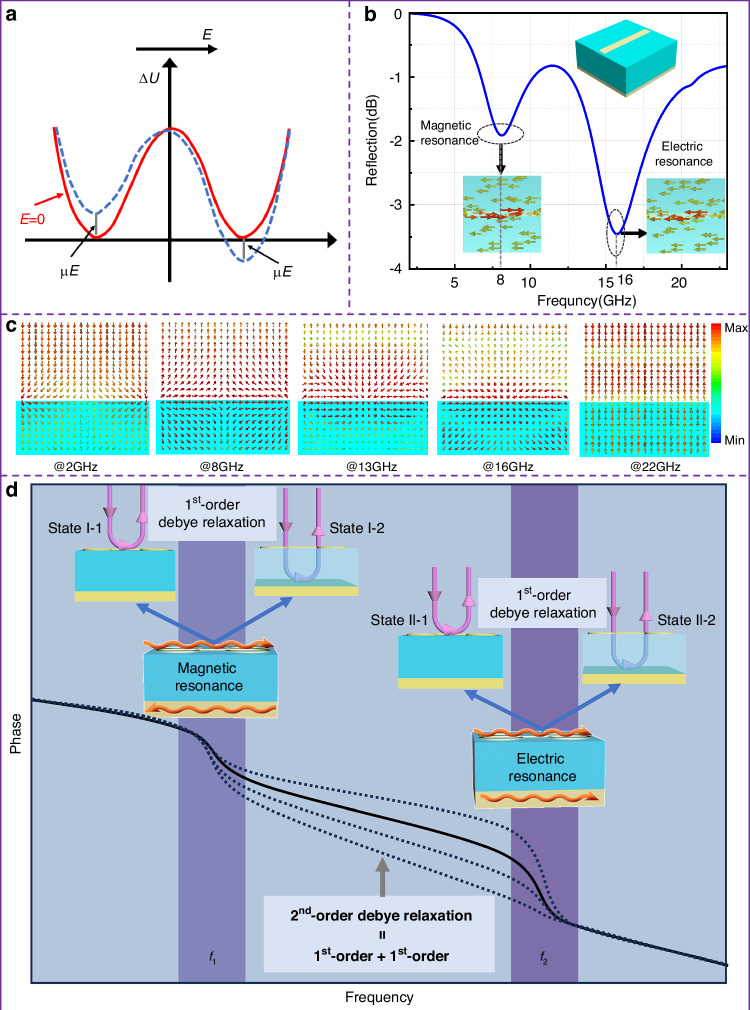
Two fundamental resonance modes of EM waves incident upon a typical reflective metasurface composed of short-wire structures. **a** The double-well model was used to interpret the relaxation process of dipole orientation polarization in conventional dielectric materials. **b** Reflection spectrum of a typical reflective metasurface composed of short-wire structures (the two lower insets show the surface currents on the short wire and background at the two resonant frequencies), where the short wire can be treated as an electric dipole since its electric size is about half a wavelength. **c** The power flows of EM waves upon the metasurface at different frequencies. **d** The schematic illustration of the 1st- and 2nd-order relaxations. Each of the two resonance modes(I: Magnetic resonance, II: Electric resonance) has two distinct states, that is, reflections upon the upper and lower interfaces. The two states can be vividly analogous to the two wells in the double-well model and enable the 1st-order relaxation process under each resonance mode. Furthermore, the two resonance modes (at *f*_1_ and *f*_2_) again form a 2nd-order relaxation process, which spans a much wider frequency band compared with that of the 1st-order relaxation processes. By modifying the 2nd-order relaxation process induced by two resonances, the phase variation between *f*_1_ and *f*_2_ can be controlled, enabling dispersion engineering

For dipole orientation polarization of traditional dielectrics, the double-well model is usually adopted to interpret the relaxation process intuitively at the microscopic level. The orientations of the dipole include at least two typical states, one of which is along external electric fields and the other perpendicular to external electric fields, corresponding to the two wells. The dipole needs to “tunnel through” the barrier between the two wells (denoted by Δ*U*). If the dipole absorbs enough energy so that its energy is higher than that of the barrier, the dipole can make a transition from one well to its neighbor. Such a transition between the two states forms the relaxation process because it takes a comparatively long time to finish one cycle of this process, and vice versa. Therefore, to explore the relaxation process within metasurfaces, it is necessary that the EM responses of metasurfaces exhibit at least two distinct states. Figure 2b shows a typical reflective metasurface composed of a 2D array of the short-wire structure (whose electric size is about half a wavelength and can be treated as an electric dipole), where the metallic dipole short wire and the conducting sheet background are separated by a dielectric spacer. Under the illumination of EM waves, the metasurface will generate surface currents both on the short wire and conducting sheet background. According to the flow directions of surface currents on the short wire and the background, there are two fundamental modes, one of which is the electric resonance mode with symmetric surface currents and the other of which is the magnetic resonance with asymmetric surface currents. Through full-wave simulations, we find that for the meta-atom structure in Fig. 2b, the magnetic and electrical resonances occur around 8.0 and 16.0 GHz, respectively. Furthermore, owing to the presence of the conducting background, the reflective metasurface can be viewed as a semi-open structure. Consequently, two types of reflections will occur for EM waves incident upon the metasurface: one occurs on the upper interface and the other on the lower conducting ground. As is depicted in Fig. 2c, the power flows of the metasurface exhibit distinct characteristics at different frequencies. Below the magnetic resonance frequency (e.g., 2.0 GHz), the EM energy is concentrated predominantly near the short wire. In contrast, at the central frequency of magnetic resonance (e.g., 8.0 GHz) and at the central frequency of electric resonance (e.g., 16.0 GHz), the energy is predominantly confined within the dielectric substrate, exhibiting strong cavity resonance phenomena. In between the two resonance frequencies (e.g., 13.0 GHz), the energy is again concentrated near the short wire. At frequencies much larger than the electric resonance frequency, the dipoles cease to respond, leading to EM energy mainly being reflected by the background. Based on the power flows, we find that below the resonance frequencies (e.g., 2.0 and 13.0 GHz), the EM field energy is mainly concentrated near the dipole surface. At the resonance frequencies (e.g., 8.0 and 16.0 GHz), the energy is mainly concentrated inside the cavity between the short wire and the background. This means that both of the two fundamental resonance modes will experience two distinct reflection states as frequency increases, as depicted in Fig. 2d. The transition between the two reflection states initiates a relaxation process. Intriguingly, the relaxation process of the metasurface resembles that of polarization relaxation of dielectrics, known as 1st-order Debye relaxation, attributed to the presence of Lorentz resonances. For a more in-depth understanding of the 1st-order Debye relaxations, please refer to S1 in Supporting Materials.

### 2nd-order Debye relaxations in metasurfaces

Considering that the metasurface can be treated as a homogeneous material and its properties can be described by the effective refractive index

1 $${{n}}^{\ast }={n}+{j}\kappa =\sqrt{{{\varepsilon }}_{{\rm {r}}}^{\ast }{{\mu }}_{{\rm {r}}}^{\ast }}$$

where *n* and *κ* are the real and imaginary parts of the effective refractive index, respectively; ε*_r_ and μ*_r_ are the effective permittivity and permeability of the metasurface, respectively. In response to incident EM waves, the metasurface will generate magnetic resonance and electric resonance. The former produces Lorentzian-type effective permeability while the latter produces Lorentzian-type effective permittivity, which will both result in 1st-order Debye-relaxation processes of reflection phases versus frequency (more details of this derivation can be found in S2 of the Supporting Materials). Specifically, considering the configuration of the meta-atom, there are usually two fundamental resonant modes in a single meta-atom, and the magnetic resonance frequency is lower than the electric resonance frequency. According to dielectric physics, the 2nd-order Debye dispersion is a superposed relaxation process given by the summation of two separate 1st-order Debye models. The corresponding parameters are the two relaxation times as well as both epsilon static values. It naturally indicates that, for a practical meta-atom, a 2nd-order relaxation process will typically be generated due to the coexistence of electric and magnetic resonances within the structure. Consequently, the 2nd-order relaxation process usually spans a quite wide band in the spectrum and is hence more important in reality, e.g., for wideband dispersion engineering. Corresponding to the 2nd-order relaxation process, the complex refractive index of the metasurface can be expressed as

2 $${{n}}^{{\ast }}=\sqrt{{{\varepsilon }}_{{{\rm {r}}}}^{{\ast }}{{\mu }}_{{\rm {r}}}^{{\ast }}}=\sqrt{{{\varepsilon }}_{{\rm{b}}}}\sqrt{{1}+{{\chi }}_{{\rm {m}}}^{\ast }}\sqrt{{1}+{{\chi }}_{{\rm{e}}}^{{\ast }}}\,\approx \sqrt{{{\varepsilon }}_{{\rm {b}}}}\left({1}+\frac{{\chi }^{{\prime} }_{{m}}}{{2}}+\frac{{j}{\chi }^{{\prime\prime} }_{{\rm {m}}}}{{2}}\right)\left({1}+\frac{{{\chi }}_{{\rm {e}}}^{\prime} }{{2}}+\frac{{j}{{\chi }}_{{\rm {e}}}^{\prime\prime} }{{2}}\right)$$

Here, *χ*_m_ and *χ*_e_ are the magnetic and electric susceptibility, respectively; ′ and *″* represent the real and imaginary part, respectively; *ε*_b_ is the permittivity of the dielectric substrate. Neglecting higher-order terms, the real part of the effective refractive index is evaluated as

3 $$n\approx \sqrt{{{\varepsilon }}_{{\rm {b}}}}\left({1}+\frac{1}{2}\frac{{{\omega }}_{{\rm {p}}{\rm {m}}}^{{2}}({{\omega }}_{0{\rm {m}}}^{{2}}-{{\omega }}^{{2}})}{({{\omega }}_{0{\rm {m}}}^{{2}}-{{\omega }}^{{2}})^{{2}}+{{\gamma }}_{{\rm {m}}}^{{2}}{{\omega }}^{{2}}}\right)+\sqrt{{{\varepsilon }}_{{\rm{b}}}}\left(\frac{1}{2}\frac{{{\omega }}_{{\rm {p}}{\rm {e}}}^{{2}}({{\omega }}_{0{\rm {e}}}^{{2}}-{{\omega }}^{{2}})}{{({{\omega }}_{0{\rm {e}}}^{{2}}-{{\omega }}^{{2}})}^{{2}}+{{\gamma }}_{{\rm {e}}}^{{2}}{{\omega }}^{{2}}}\right)$$

Here, *ω*_pm_ and *ω*_pe_ are the plasma frequencies of the magnetic and electric resonances, respectively; *ω*_0m_ and *ω*_0e_ represent the magnetic and electric resonance frequencies, respectively. The refractive index formula is composed of two resonant processes. Since each resonance corresponds to a 1st-order Debye relaxation, the refractive index naturally exhibits a 2nd-order Debye relaxation process, and the infinite value of the first relaxation is the static value of the second relaxation process. By examining the energy flow density in Fig. 2c, it can be observed that at 22 GHz, the microstructure has little effect, and the electromagnetic wave directly passes through the dielectric substrate and is reflected by the metallic sheet, exhibiting the behavior of a pure substrate, thus indicating that the effective permittivity of the metasurface is equal to the substrate. This implies that the refractive index (static value of the second relaxation process) at high frequencies is $\sqrt{{\varepsilon }_{b}}$. Therefore, we further simplify Eq. (3) into the following form:

4 $$\begin{array}{l}n=\frac{{n}_{1{\rm {s}}}-{n}_{{\rm {1}}\infty }}{1+{\tau }_{1}^{2}{\omega }^{2}}+{n}_{{\rm {2}}\infty }+\frac{{n}_{{2s}}-{n}_{2\infty }}{{1}+{\tau }_{{2}}^{{2}}{\omega }^{{2}}}\\ \left\{\begin{array}{l}{n}_{{2}\infty }=\sqrt{{\varepsilon }_{{\rm {b}}}}\\ {n}_{{2s}}={n}_{{2}\infty }\left({1}+\frac{{\omega }_{{\rm {pe}}}^{2}}{2({\omega }_{0{\rm {e}}}^{2}-{\omega }^{2})}\right)\\ {\tau }_{2}=\frac{{\gamma }_{{\rm {e}}}^{2}}{{\omega }_{{0}{\rm {e}}}^{2}-{\omega }^{2}}\end{array}\right.\left\{\begin{array}{l}{n}_{{1}\infty }={n}_{{2s}}\\ {n}_{{1s}}={n}_{{2}\infty }\left({1}+\frac{{\omega }_{{\rm {pe}}}^{2}}{2({\omega }_{0{\rm {e}}}^{2}-{\omega }^{2})}+\frac{{\omega }_{{\rm {pm}}}^{2}}{{2}({\omega }_{0{\rm {m}}}^{2}-{\omega }^{2})}\right)\\ {\tau }_{{1}}=\frac{{\gamma }_{{\rm {m}}}^{2}}{{\omega }_{0{\rm {m}}}^{{2}}-{\omega }^{{2}}}\end{array}\right.\end{array}$$

The reflection phase of the metasurface can be expressed as

5 $$\begin{array}{l}\varphi ({\omega })=2{k}_{0}{{d}}n=\frac{{k}_{0}{{d}}\sqrt{{{\varepsilon }}_{{\rm {b}}}}}{2}\left(1+\frac{{{\omega }}_{{\rm {p}}{\rm {m}}}^{{2}}({{\omega }}_{0{\rm{m}}}^{{2}}-{{\omega }}^{{2}})}{{({{\omega }}_{0{\rm {m}}}^{{2}}-{{\omega }}^{{2}})}^{{2}}+{{\gamma }}_{{\rm {m}}}^{{2}}{{\omega }}^{{2}}}\right)+\frac{{k}_{0}{\rm {d}}\sqrt{{{\varepsilon }}_{{\rm {b}}}}}{2}\left(\frac{{{\omega }}_{{\rm {p}}{\rm {e}}}^{{2}}({{\omega }}_{0{\rm {e}}}^{{2}}-{{\omega }}^{{2}})}{{({{\omega }}_{0{\rm {e}}}^{{2}}-{{\omega }}^{{2}})}^{{2}}+{{\gamma }}_{{\rm {e}}}^{{2}}{{\omega }}^{{2}}}\right)=\frac{{\varphi }_{1{s}}-{\varphi }_{{1}\infty }}{{1}+{\tau }_{1}^{2}{\omega }^{2}}+{\varphi }_{{2}\infty }+\frac{{\varphi }_{2{s}}-{\varphi }_{2\infty }}{{1}+{\tau }_{{2}}^{{2}}{\omega }^{{2}}}\\ \left\{\begin{array}{l}{\varphi }_{{2}\infty }=2{k}_{0}{{d}}\sqrt{{\varepsilon }_{{{b}}}}\\ {\varphi }_{2{s}}={\varphi }_{{2}\infty }\left({1}+\frac{{\omega }_{{{pe}}}^{2}}{{2}({\omega }_{0{{e}}}^{2}-{\omega }^{2})}\right)\\ {\tau }_{2}=\frac{{\gamma }_{{{e}}}^{2}}{{\omega }_{{0}{{e}}}^{2}-{\omega }^{2}}\end{array}\right.\left\{\begin{array}{l}{\varphi }_{{1}\infty }={\varphi }_{2{s}}\\ {\varphi }_{1{s}}={\varphi }_{{2}\infty }\left({1}+\frac{{\omega }_{{{pe}}}^{2}}{2({\omega }_{0{{e}}}^{2}-{\omega }^{2})}+\frac{{\omega }_{{{pm}}}^{2}}{{2}({\omega }_{0{{m}}}^{2}-{\omega }^{2})}\right)\\ {\tau }_{{1}}=\frac{{\gamma }_{{{m}}}^{2}}{{\omega }_{0{{m}}}^{{2}}-{\omega }^{{2}}}\end{array}\right.\end{array}$$

where *d* is the thickness of the metasurface, *k*_0_ is the wave number in free space. The above analysis indicates that each resonance can generate one 1st-order relaxation process of reflection phase variation, and the metasurface can form a 2nd-order relaxation process by combining the magnetic and electric resonances, as is shown in Fig. 2d. Considering that each resonance will bring a phase relaxation around the resonant frequency, the phase variation between the two resonances is quite smoothy and approximately linearly versus frequency. By changing the parameters of the two resonances, including resonance frequencies and resonance strengths, the slope of the phase profile can be customized according to our will, which enables a high degree of freedom for wideband dispersion engineering.

## Discussion

In the subsequent discussion section, we investigated the design of broadband dispersion control of metasurfaces based on second-order relaxation theory, exemplified by a focusing metasurface for simulation and experimental validation.

### Meta-atom with 2nd-order Debye relaxation of reflection phase

**Fig. 3:**
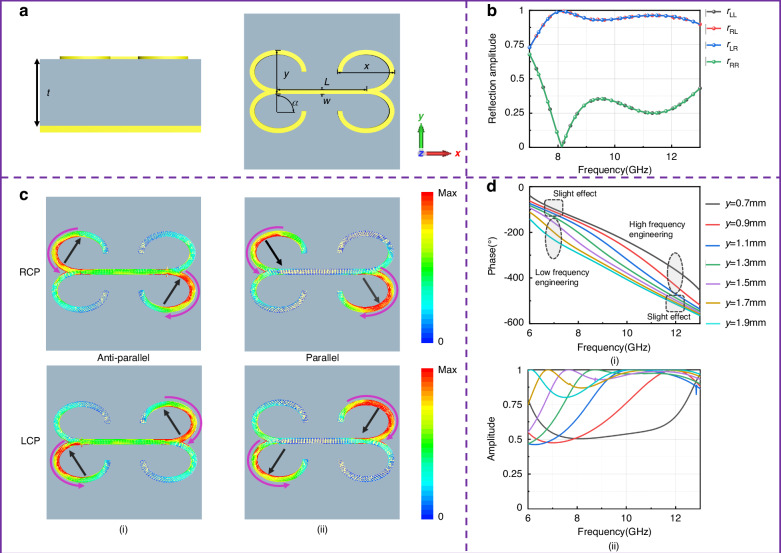
The proposed meta-atom with 2nd-order Debye relaxation for wideband phase tailoring. **a** Schematic illustration of the QEA meta-atom. **b** the reflection amplitude under RCP and LCP wave illumination. **c** the surface currents on the structure and ground sheet at (i) 7.0 GHz and (ii) 12.0 GHz. The top row is the surface currents induced by the RCP wave illumination, while the bottom row is the surface currents induced by the LCP wave illumination. **d** the phase (i) and amplitude (ii) response to the different *y* under RCP wave illumination

As a proof-of concept, a quadru-elliptic-arc (QEA) structure is proposed as the meta-atom for metasurface design to demonstrate the powerful capability of wideband dispersion engineering based on 2nd-order Debye relaxations. The schematic diagram of the meta-atom is depicted in Fig. 3a, comprising one thin layer of QEA and one background sheet separated by a dielectric spacer with a thickness of 4.0 mm. The metallic ground sheet, the F4B dielectric (*ε*_r_ = 2.65, tan *δ* = 0.001) spacer and the QEA structure layer are stacked one by one from the bottom to the top. Through full-wave simulations, we obtained the reflection performance of the meta-atom under the illumination of circularly-polarized (CP) waves (more details of the simulation see the subsection “simulation”, in the “Materials and methods” section), as is plotted in Fig. 3b. It should be noted that CP illumination is specially selected here because CP waves can excite rotation-like movements of free electrons within the metallic structures, which can mimic vividly the orientation polarization in dielectric physics. The *r*_LR_ and *r*_RR_ are the cross- and co-polarization components under the illumination of right-handed circularly polarized (RCP) waves, respectively; *r*_RL_ and *r*_LL_ are the cross- and co-polarization components under the illumination of left-handed circularly polarized (LCP) waves, respectively. It can be found that the QEA meta-atom exhibits high-efficiency polarization conversion performance across the entire X band from 8.0 to 12.0 GHz. Due to the mirror symmetry of the meta-atom, *r*_RL_ and *r*_LR_ have identical reflection phases and amplitude. The wideband performance is achieved due to the large frequency span of the 2nd-order Debye relaxation formed by two resonance modes (magnetic and electric resonances). Surface currents on the QEA structure at 8.0 and 12.0 GHz are monitored, as shown in Fig. 3c, where the purple and black arrows represent the directions of currents on the QEA structure and the metallic ground sheet, respectively. It can be found that the currents are mainly concentrated on the elliptic arc at 8.0 GHz, and the surface currents on the structure and the ground sheet flow in opposite directions, indicating a magnetic resonance. At 12.0 GHz, the surface currents on the structure and the ground sheet flow in the same direction, indicating an electric resonance. The two resonances together form a 2nd-order Debye relaxation process which spans quite wide in the frequency band. This lays a solid foundation for wideband dispersion engineering of metasurfaces composed of the QEA structure meta-atom.

Based on the 2nd-order Debye relaxation of the QES meta-atom, the dispersion within 8.0–12.0 GHz can be tailored by adjusting the structural parameters of elliptical arcs. To this end, the reflection phase and amplitude of *r*_LR_ response to different *y-*diameters are plotted in Fig. 3d to demonstrate the dispersion engineering. Considering the proposed meta-atom design lacks charity, left-hand circularly polarized waves exhibit the same response characteristics as right-hand circularly polarized waves upon illumination. As such, we solely present the analysis and explanation of the unit cell reflection performance under right-hand circularly polarized wave incidence. It can be seen from Fig. 3d that the phase dispersion can be tailored by high-frequency resonance (at 11.0–13.0 GHz) and low-frequency resonance (at 6.0–8.0 GHz). For *y* $\in$[0.7,1.2 mm], the phase variation slope in the higher frequency band is steeper, while that in the lower frequency band is more gradual. On the contrary, for *y* $\in$[1.5,1.9 mm], the phase response in the lower frequency band is steeper, while that in higher frequency band is moderate. In terms of amplitude variation, it can be observed that while there is fluctuation in the amplitudes, apart from specific outliers, the amplitudes corresponding to the remaining parameters are consistently high (with the lowest value still above 0.5), thereby meeting the design requirements. Figure S2 in Supporting Information presents the phase and amplitude responses under variations of other parameters. These results collectively demonstrate that the 2nd-order Debye relaxation plays a pivotal role in wideband engineering dispersion, which can change the slope of phase variations and expand the range of phase spans. Through systematic parameter adjustments (such as *α*, *L*, *y*, and *x*), a meta-atoms library comprising 7310 variations of the QEA structure meta-atom with different phase responses was constructed.

**Fig. 4:**
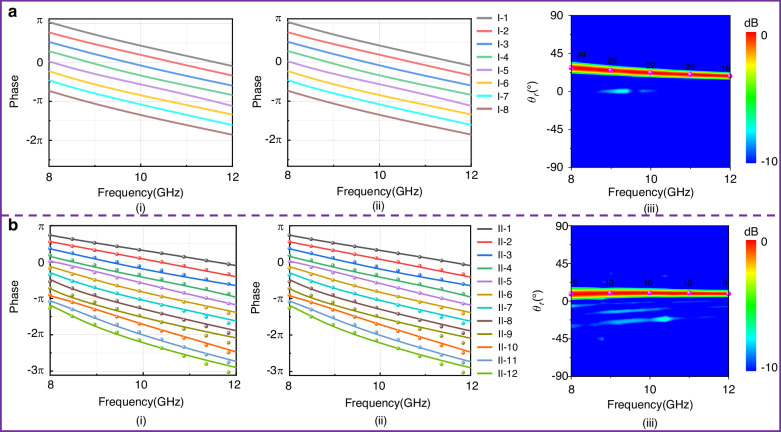
Phase responses of two sets of meta-atoms selected from the meta-atom library and the corresponding normalized far-field patterns of reflective metasurfaces composed of the two sets of meta-atoms. **a** Parallel phase lines of one set of 8 meta-atoms for forming constant phase gradient in X-band: (i) *r*_RL_, (ii) *r*_LR_, (iii) the corresponding normalized far-field pattern. **b** phase responses of the other set of 12 meta-atoms for forming achromatic phase gradient: (i) *r*_RL_, (ii) *r*_LR_, (iii) the corresponding normalized far-field pattern, where the colored dots in panels (i) and (ii) represent the theoretically predicted phases for achromatic deflection

The required phase dispersion of the metasurface can be arranged by selecting the corresponding meta-atoms from the library. We firstly demonstrate two wideband beam deflectors with 1D phase gradient as a proof-of-concept, one of which is with constant phase gradient (equivalent to constant additional wave-vector **k**_||_ which is tangential to the metasurface) and the other of which with linearly increasing phase gradient (equivalent to achromatic additional wave-vector **k**_||_ which is tangential to the metasurface). The phase variation of the metasurfaces forms a gradient along *x* direction while remains constant in *y* direction. According to the generalized Snell’s reflection law, the deflected angle is calculated as^11,29^

6 $${\rm {s}}{\rm{i}}{\rm{n}}{{\theta }}_{{{r}}}=\frac{{c}}{{f}}\frac{{\mathbf{\Delta }}{\varphi }({f})}{{2}{\pi }{P}}$$

where *P* is the repeating period of the meta-atom, *f* is the operation frequency, *c* is the velocity of light in vacuum, ∆*φ*(*f*) the phase step between each two adjacent meta-atoms. As is depicted in Fig. 4a, eight meta-atoms with the desired linear phase distribution are selected to compose a super-cell. The parameters and schematic diagram of the selected meta-atoms are listed in S3 of Supporting Information. It can be seen that the desired linear phase distribution with a phase step ∆*φ* = 45° covers the entire X band from 8.0 to 12.0 GHz. It should be noted that the phase response is totally different from P-B phase since we do not rotate the meta-atoms, and the phase imparted by the meta-atoms to LCP and RCP waves is identical (*φ*_L_ = *φ*_R_), rather than opposite in sign (*φ*_L_ = −*φ*_R_). A beam deflection metasurface (named metasurface I) composed of 8 meta-atoms is implemented. Considering that the phases imparted to LCP and RCP waves are identical, we just need to calculate normalized far-field patterns under LCP or RCP waves (here, we select RCP waves) to demonstrate the performance, as shown in Fig. 4a-iii. Normally incident waves are deflected to an angle of *θ*_r_. When ∆*φ*(*f*) is constant, the deflection angle *θ*_r_ gradually decreases as the frequency increases, in good agreement with the theoretical predictions.

To demonstrate the powerful capacity of wideband dispersion engineering, 12 meta-atoms with phase variations for achromatic deflection are further selected from the library, and the phase responses are plotted in Fig. 4b. The colored dots represent the theoretically predicted phases for achromatic deflection. Similarly, those meta-atoms are arranged in sequence to compose a metasurface II with the same areas as metasurface I. The parameters and schematic diagram of the selected meta-atoms are listed in S3 of Supporting Information. For metasurface II under RCP wave illumination, the normalized far-filed pattern is also plotted in Fig. 4b-iii. It can be seen that the reflected waves are deflected in the same direction within the entire X band of 8.0–12.0 GHz due to linearly increasing phase step versus frequency, which is different from metasurface I. Furthermore, the undesired orders of diffraction beams are evidently suppressed, with an order of magnitude lower energy than the main beam. Thus, the performance ensures high efficiency of beam deflection.

### Wideband planar focusing metasurfaces

**Fig. 5:**
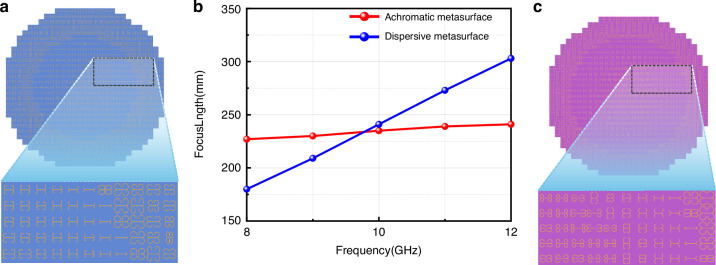
Simulated results under CP wave illumination on y-o-z plane. **a** The diagram of achromatic focusing metasurface. **b** The focus length variations versus frequency. **c** The chromatic focusing metasurface

In the above section, the dispersion with constant phase gradient and achromatic phase gradient are realized via the QEA structure meta-atom. Herein, two planar-focusing metasurfaces with 2D phase profiles, one for chromatic focusing and the other for achromatic focusing, are implemented. For a set of meta-atoms with parallel phase lines versus frequency, the phase gradient keeps constant with varying frequency, so the focal length of such a metasurface will be lengthened as frequency increases. In contrast, achromatic phase response means that the phase gradient is linearly proportional to frequency rather than a constant. This requires that the phase dispersion slope be tailorable so that each dispersion line of the selected set of meta-atoms has different slopes. The phase of the meta-atoms located in the coordinates (*x*, *y*) can be calculated by^34,35^

7 $$\left\{\begin{array}{l}{{\varphi }}_{1}({x},{y})=\frac{{2}{\pi }{f}}{{c}}(\sqrt{{{x}}^{{2}}+{{y}}^{{2}}+{{F}}^{{2}}}-{F})+{\varphi }(0,0),\\ {{\varphi }}_{2}({x},{y},{f})=\frac{{2}{\pi }{f}}{{c}}(\sqrt{{{x}}^{{2}}+{{y}}^{{2}}+{{F}}^{{2}}}-{F})+{\varphi }(0,0,{f})\end{array}\right.$$

where *φ*_1_(*x*, *y*) is the phase distribution for chromatic focusing, *φ*_2_(*x*, *y*, *f*) the phase distribution for achromatic focusing, *φ*(0, 0) and *φ*(0, 0, *f*) the reference phase of the meta-atom at the center position, *f* the operating frequency*, x* and *y* the coordinates of meta-atoms in the *x*-direction and *y*-direction, respectively; *F* is the designed focus length and is set to be 300 mm at 10.0 GHz. The meta-atoms that satisfy Eq. (6) are selected from the meta-atom library to construct the two 2D focusing metasurfaces, as is shown in Fig. 5a.

For normally incident CP waves in 8.0–12.0 GHz, the reflected waves upon the chromatic metasurface will be focused to diverse locations at different frequencies, while the achromic metasurface will focus reflected waves to the same location in the entire X band. Through full-wave simulations, the transverse electric field intensity distributions (|*E*_||_|^2^ = |*E*_*x*_|^2^ + |*E*_*y*_|^2^) and the focus positions under CP wave illumination are obtained and depicted in Fig. 5a, b. The blue dashed line represents the focus plane (*z* = 300 mm) of the designed metasurface. For the chromatic focusing metasurface, EM waves will be focused. However, the focus spot location will change with frequency. By calculating the focal positions, we find that the focal length is between 180 and 303 mm from 8.0 to 12.0 GHz. While for the achromatic focusing metasurface, the focus location remains almost unchanged. Further calculations indicate that the focal length is between 227 and 241 mm from 8.0 to 12.0 GHz, which verifies the achromatic performance. Compared with the chromatic focusing metasurface, the variation of focal position with frequency is significantly suppressed. It can also be seen that the focal length obtained by simulation is 60 mm shorter than the designed result for both chromatic and achromatic metasurfaces. This mainly can be ascribed to the mutual couplings between the selected meta-atoms that lead to the discrepancy. Besides, The focusing efficiency of the focus metasurface is calculated, which is defined as ∑*E*_f_^2^/∑*E*_i_^2^, where ∑*E*_f_^2^ is the reflected electric field energy of the focal spot and ∑*E*_i_^2^ is the electric field energy of the incident wave^35^. we analyze the case of the RCP wave incident on the achromatic focusing metasurface and compute the electric field distribution data at the focal plane. Following the definition, we calculate efficiencies at 8, 10, and 12 GHz to be 41.44%, 48.27%, and 37.04%, respectively, further validating the focusing performance of our metasurface. Considering that linear- and elliptical-polarization waves can be decomposed into LCP and RCP components, the same phase obtained by the CP components will be imparted to the synthetic linear- and elliptical-polarization waves. Therefore, the two metasurfaces can still achieve chromatic and achromatic focusing for linear- and elliptical-polarization waves (more details can be found in S4 of Supporting Information).

**Fig. 6:**
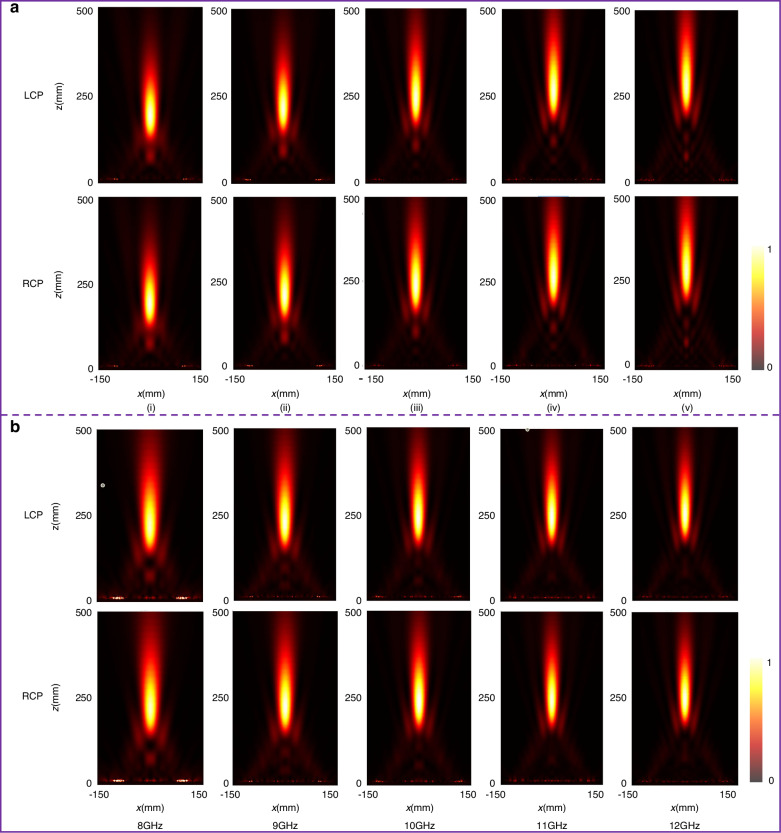
The simulated electric field intensity. **a** Simulated electric field intensity distribution (|*E*_||_|^2^ = |*E*_*x*_|^2^ + |*E*_*y*_|^2^) for the chromatic focusing metasurface: (i) 8.0 GHz, (ii) 9.0 GHz, (iii) 10.0 GHz, (iv) 11.0 GHz, (v) 12.0 GHz. **b** Simulated electric field intensity distribution (|*E*_||_|^2^ = |*E*_*x*_|^2^ + |*E*_*y*_|^2^)for the achromatic focusing metasurface: (i) 8.0 GHz, (ii) 9.0 GHz, (iii) 10.0 GHz, (iv) 11.0 GHz, (v) 12.0 GHz

**Fig. 7:**
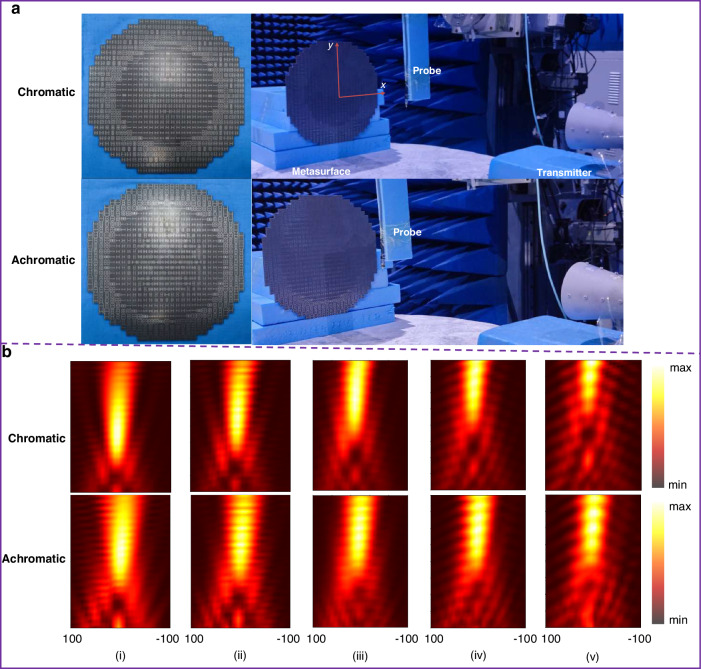
The near-field measured results of electric field distributions. **a** The prototypes and measured setup. **b** The measured result of electric field intensity distributions (|*E*|||^2^ = |*E*_*x*_|^2^ + |*E*_*y*_|^2^) under RCP wave illumination at *y-o-z* plane: (i) 8.0 GHz, (ii) 9.0 GHz, (iii) 10.0 GHz, (iv) 11.0 GHz, (v) 12.0 GHz

To further demonstrate the focusing performance, we fabricated two prototypes of the same size. Near-field measurements were conducted in microwave anechoic chamber. Figure 7a shows the measurement setup, where a CP antenna is placed in the far-field as a feed source, and a near-field probe is placed closely in front of the metasurface to scan the *y-o-z* plane (more details of the measurements see the subsection “experiments”, in the “Materials and methods” section). Considering the metasurfaces have the same performance under LCP and RCP wave illumination, the transverse electric field intensity distribution under RCP wave illumination at *y-o-z* plane are depicted in Fig. 6b. It can be seen from the top panel of Fig. 6b, that the |*E*_||_|^2^ is mainly concentrated in the central region, and the focus position moves up gradually with the increase of frequency. On the contrary, the |*E*_||_|^2^ of the achromatic metasurface depicted in the bottom panel indicated that the focus position remains unchanged. The measured results indicated that the two metasurfaces achieve chromatic and achromatic focusing in the entire X-band from 8.0 to 12.0 GHz, respectively, in good agreement with the simulations. The slight discrepancy between simulation and measurement results is mainly because the probe is connected to a vector network analyzer through the RF cable assemblies, and the RF cable assembly will inevitably appear in the scanning area in front of the metasurface.

Debye relaxation is one of the most important physical processes in dielectric physics. In this work, we firstly show, both by theoretical prediction and by experimental verification, that the phase responses of metasurfaces under resonances experience 1st-order Debye relaxation process versus frequency, although these resonances are typically Lorentzian type. More intriguingly, by combining multiple resonance modes with 1st-order Debye relaxation of phase responses, higher-order (e.g., 2nd-order) Debye relaxations can be also devised, which can span quite a wide frequency band in the spectrum. This opens up a new route to dispersion engineering in a wide band, which is very important in designing wideband, multi-functional, or multiplexed devices or systems using metasurfaces. To demonstrate the powerful capacity of wideband dispersion engineering based on higher-order Debye relaxations, we propose a meta-atom structure with 2nd-order Debye relaxation formed by two adjacent magnetic and electric resonances, and establish a meta-atom library based on this meta-atom. Using this meta-atom, we implemented two metasurface deflectors with constant phase gradient and linearly-increasing phase gradient with frequency and further demonstrated experimentally two planar focusing metasurfaces with chromatic and achromatic focus lengths, which convincingly verify the powerful capability of wideband dispersion engineering based on higher-order Debye relaxations. This work explores Debye relaxations in metasurfaces for wideband dispersion engineering and provides a new understanding of resonances in metamaterials, which can be readily extended to other frequency regimes, e.g., terahertz and even optical frequencies.

## Materials and methods

### Simulation

All of the simulations are implemented by CST Microwave Studio. With the help of the CST field monitor, the surface current and electric field distribution can be calculated and depicted in manuscript. For the meta-atom simulation, the boundary conditions along the *x* and *y* directions are set as the “unit cell” while two Floquet ports are fixed along the z-directions. The plane wave with the corresponding polarization state is illuminated from the +*z* to −*z* direction. Frequency Domain Solver is utilized to conduct simulation. While for the array simulation, the boundary conditions along the *x*, *y*, and *z* directions are set as the “Open Add Space” and “Time-Domain Solver” is used.

### Establishment and screening of the meta-atom library

In the meta-atom simulation, electromagnetic resonances respond to parameters *L*, *α*, *x*, and *y*. Therefore, with the assistance of the CST parameter scanning module, a meta-atom library containing 7310 elements was established through simulations of variations in the above parameters. Furthermore, in order to search for elements that satisfy chromatic and achromatic focusing, a Matlab program was developed with Eq. (6) as a constraint, selecting meta-atoms from the library with reflection phases that meet the criteria, and assembling a functional metasurface.

### Experiment

The metasurface is fabricated by the printed circuit board (PCB) All the experiment are conducted in the microwave anechoic. For the near-field measure, the measured state is illustrated in Fig. 7a. The transmitting antenna is placed far away from the metasurface to ensure that the wave incident on the metasurface is approximately plane wave. The monopole antenna is used as the probe to scan the electric field distribution in the *y*-*o*-*z* plane.

## Supplementary information

Supplementary Information for 2nd-Order Debye Relaxation in Electromagnetic Metasurfaces for Wideband Dispersion Engineering

## Data Availability

Data will be available upon request.
