# Supplementary material for: 2nd-Order Debye relaxation in electromagnetic metasurfaces for wideband dispersion engineering
Source: Light Sci Appl. 2025 Mar 27;14:143. doi: 10.1038/s41377-025-01813-1 (PMC11950317; doi:10.1038/s41377-025-01813-1)
Supplement: Supplementary file 1 — Supplementary Information for 2nd-Order Debye Relaxation in Electromagnetic Metasurfaces for Wideband Dispersion Engineering [file 41377_2025_1813_MOESM1_ESM.docx]

**Supporting Materials**

**2nd-Order Debye Relaxation in Electromagnetic Metasurfaces for Wideband Dispersion Engineering**

Xinmin Fu^1^^†^, Yajuan Han^1,2†^, Jiafu Wang^1,2^*, Jie Yang^1^*, Yong Sun^1^, Chang Ding^1,2^, Yuxiang Jia^1,2^, Jun Wang^1^, Shaobo Qu^1^, Tiejun Cui^3^*

^1^*Shaanxi Key Laboratory of Artificially-Structured Functional Materials and Devices, Air Force Engineering University, Xi’an 710051, China*

*^2^Suzhou Laboratory, Suzhou Jiangsu 215000, China*

*^3^Institute of Electromagnetic Space, Southeast University, Nanjing, 210096, China*

*E-mail: wangjiafu1981@126.com

*E-mail: yangjie_phy@163.com

*E-mail: tjcui@seu.edu.cn

^†^These authors contributed equally to this work

**S1: The Debye relaxation**

**1. The polarization mechanisms and Debye relaxation**

Considering thatmetamaterials/metasurfaces, as an artificial structural and functional material, is composed of discrete metal structures and dielectric substrates, it can be regarded as a special dielectric material. Therefore, it is necessary for us to understand the fundamentals of dielectric polarization and permittivity. Polarization is a primary electrical property of dielectric materials under an electric field, characterized by the phenomenon of charge migration. It involves the displacement of charges (including bound charges within molecules or regions where charges cannot freely move, as well as free charges) in dielectric materials under an applied electric field, leading to small generalized displacements (such as the displacement of bound charges and orientation of dipoles) or restricted large-scale displacements (such as the movement of free charges to interfaces and electrode surfaces), resulting in bound charges at the surfaces (or interfaces) of dielectric materials and the induction of physical processes for dipole moments. As shown in Fig S1, Common polarization mechanisms in dielectrics mainly include four types: electronic polarization, dipole orientation polarization, ionic polarization, and interface polarization. (a) Electronic polarization occurs in the frequency range of 10^14-10^16 Hz, where polarization arises due to atomic positions relative to the atomic nucleus changing, leading to the formation of dipole moments, which is a universal phenomenon for all materials. (b) Ionic polarization occurs within the frequency range of 10^9-10^13 Hz, and it is caused by relative displacement of cations and anions. (c) Dipole orientation polarization takes place within the frequency range of approximately 10^3-10^8 Hz, also known as orientation polarization, resulting from the presence of an unbalanced charge distribution in atoms or ions, where these charges (dipoles) align parallel to the electric field direction, creating what is known as orientation polarization. (d) Space charge polarization, occurring within the frequency range of 10^-3-10^2 Hz, also known as interfacial polarization, is caused by movable charges or charges from different electrodes being obstructed by interfaces or confined within materials. The frequency-dependent variation of the permittivity is referred to as the dispersion of dielectrics. Fig S1 illustrates that dispersion resulting from different polarization mechanisms manifests as resonant-type dispersion and relaxational-type dispersion. Ion polarization and electronic polarization mechanisms exhibit resonant-type dispersion, analyzed by harmonic oscillator motion, while dipole reorientation polarization and interface polarization exhibit relaxational-type dispersion, analyzed using the Debye model. In fact, the reflection of light by metallic thin films reflects the response of metals to optical frequency electric fields. The generation of the dielectric constant of metals mainly arises from the collective oscillation of free electrons in metals, typically analyzed using the Drude model. Here, we begin by analyzing relaxational dispersion. The characteristic feature of relaxational dispersion is the decrease of the real part of the dielectric constant from one steady-state value to another with increasing frequency, while the imaginary part of the permittivity reaches a peak as the real part decreases. This change arises from the alignment of the electric dipoles parallel to the applied electric field. However, under an alternating electric field, the dipoles continuously rotate to establish equilibrium, giving rise to relaxation phenomena. The mathematical expression for dielectric relaxation is as follows[1,2]:

$\varepsilon_{r}^{*}\left(\omega\right)=\varepsilon_{\infty}+\left(\varepsilon_{s}-\varepsilon_{\infty}\right)f\left(\omega\right)$ (S1)

Where *f*(ω) is the relaxation function. *ε*_∞_ and *ε*_s_ are respectively the infinite and static permittivity limits, *ω* is the angular frequency of an external electric field change. The specific form of relaxation is determined by the relaxation function. Debye has carried on the deep research on the dielectric relaxation process, and puts forward the debye relaxation [1,2]:

$\left\{\begin{matrix} \varepsilon_{r}^{*}=\varepsilon'_{r}+j\varepsilon''_{r}=\varepsilon_{\infty}+\frac{\varepsilon_{s}-\varepsilon_{\infty}}{1+j\omega\tau} \\ \varepsilon'_{r}=\varepsilon_{\infty}+\frac{\varepsilon_{s}-\varepsilon_{\infty}}{1+\omega^{2}\tau^{2}} \\ \varepsilon''_{r}=\frac{\left(\varepsilon_{s}-\varepsilon_{\infty}\right)\omega\tau}{1+\omega^{2}\tau^{2}} \end{matrix}\right.$ (S2)


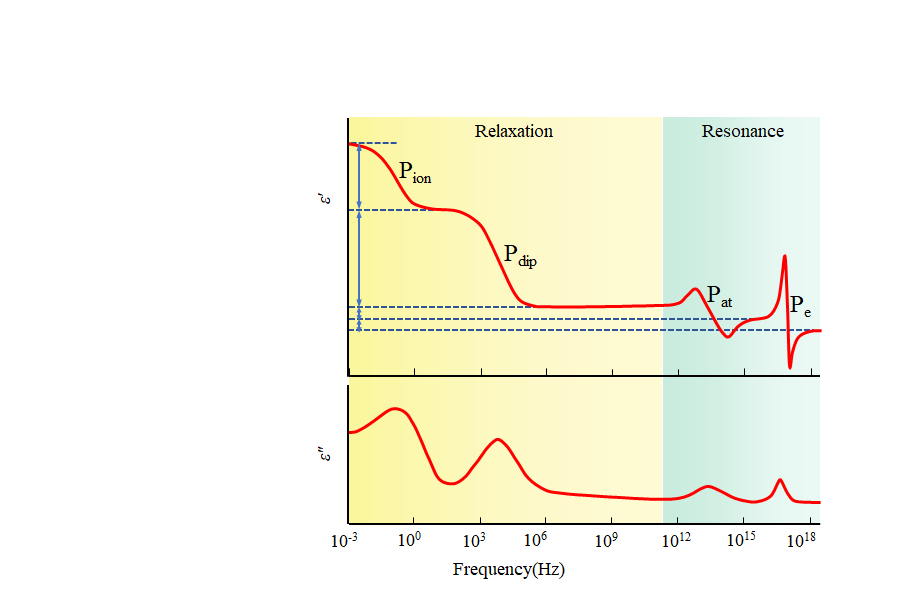


Fig. S1 The permittivity as a function of frequency in dielectric under different type of polarization[10]. P_ion_: ionic polarization, P_dip_: dipole orientational polarization, P_at_: atomic polarization, P_e_: electric polarization. The top penal is the real part of the permittivity, while the bottom penal is the imaginary part.

Electronic and atomic polarizations originate from electron cloud and skeletal atom movements deviated away from the equilibrium position as induced by an external field, and thus they exhibit resonance foam and occur at very high frequencies, i.e., in the infrared and optical range. Near the resonant frequency, the real part of the permittivity first increases and then decreases sharply, and finally gradually increases and returns to the new steady-state value. At the same time, the imaginary part of the permittivity appears a peak. This process also exhibits relaxation characteristics.

**2.** Derivation process of the Debye relaxation

The variation of the complex permittivity depends on the relaxation function which in turn depends on the polarization micro-mechanism. We first analyze the establishment process of electric polarization *P*. the step electric field *E*(*t*)=*E*_0_S(*t*) is first loaded to the dielectric, after a long enough time, the maximum value of the thermal equilibrium polarization intensity *P*_re_ of the dielectric is established[3,9]:

**$P_{re}=\varepsilon_{0}\chi_{r}E_{0}$**  (S3)

Consider that the changing rate of *P* at time *t* is proportional to the difference between *P*_re_ and the electric polarization at that time. Thus[1,9]:

$\left\{\begin{matrix} \frac{dP_{r}(t)}{dt}=\frac{1}{\tau}\left(\varepsilon_{0}\chi_{r}E_{0}-P_{r}\right) \\ P_{r}\left(t=0\right)=0 \end{matrix}\right.$ (S4)

Where *χ_r_=ε_s_-ε_∞_*, 1/τ is the scale coefficient with time dimension, which is called time constant. We can obtain *P*_r_(*t*) by solving the above equation[1-3,9].

$\left\{\begin{matrix} \text{ }P_{r}\left(t\right)=\varepsilon_{0}\chi_{r}E_{0}\left(1-e^{-\frac{t}{\tau}}\right)=\left(\varepsilon_{s}-\varepsilon_{\infty}\right)\varepsilon_{0}E_{0}\left(1-e^{-\frac{t}{\tau}}\right) \\ \frac{dP_{r}\left(t\right)}{dt}=\left(\varepsilon_{s}-\varepsilon_{\infty}\right)\varepsilon_{0}E_{0}\frac{1}{\tau}\left(1-e^{-\frac{t}{\tau}}\right) \end{matrix}\right.$ (S5)

Thus, the relaxation function *f*(*t*)=(1/*τ*)*e*(-*t*/*τ*). Now, we consider the applied electric field has an alternating mode, i.e. *E*(*t*)=*E*_0_e*^iωt^*. The steady-state solution for *P*_r_(*ω*) is:

$P_{r}\left(\omega\right)=\varepsilon_{0}\left(\varepsilon_{s}-\varepsilon_{\infty}\right)\int_{0}^{\infty} f\left(y\right)E\left(t-y\right)dy=\frac{\varepsilon_{0}\chi_{r}E\left(t\right)}{1+j\omega\tau}$ (S6)

Therefore, the total electric polarization is:

$P_{r}\left(\omega,t\right)=P_{\infty}\left(t\right)+P_{r}\left(\omega\right)=\varepsilon_{0}\left(\chi_{\infty}+\frac{\chi_{r}}{1+j\omega\tau}\right)E\left(t\right)=\varepsilon_{0}\chi^{*}E\left(t\right)$ (S7)

We can further calculate the electric polarization and permittivity:

$\left\{\begin{matrix} \chi^{*}\left(\omega\right)=\chi_{\infty}+\frac{\chi_{r}}{1+j\omega\tau} \\ \varepsilon_{r}^{*}\left(\omega\right)=1+\chi^{*}\left(\omega\right)=\varepsilon_{\infty}+\frac{\varepsilon_{s}-\varepsilon_{\infty}}{1+j\omega\tau} \end{matrix}\right.$ (S8)

**S2: The relaxation behavior induced by the resonance**

Reflective metasurace, as the double metallic layer structure, has dual resonance modes, namely electric and magnetic resonance, respectively. Each resonance mode will experience two distinct reflection states as frequency increases. The transition between the two reflection states initiates a relaxation process, known as 1st-order Debye relaxation. Such a relaxation process is mainly attributed to the presence of Lorentz resonances.

**1. 1st-order Debye-relaxation induced by electric resonance**

Considering that the electric quantity per charge in the medium is *q* and there are *N* dipoles in the medium, the electric dipole moment **P** in the medium can be expressed as:

$\mathbf{P}=Nq\mathbf{r}$ (S9)

Where **r** is the distance that the charge deviates from its equilibrium position under an applied electric field. The natural resonance frequency of the dipole is assumed to be ω_0_. The equation of motion can be expressed as[5,6-9]:

$m\frac{\partial^{2}r}{\partial t^{2}}+m\gamma\frac{\partial r}{\partial t}+m\omega_{0}^{2}r=-qE$ (S10)

Consider the applied electric field **E**(*ω*) =E_0_*e^jωt^,* **r**(ω)= r_0_*e^jωt^.* Thus, the Eq. (S10) can be becomes:

$\left(-m\omega^{2}-j\omega m\gamma+m\omega_{0}^{2}\right)\mathbf{r}\left(\omega\right)=-q\mathbf{E}(\omega)$ (S11)

Thus:

$\mathbf{r}\left(\omega\right)=-\frac{q}{m}\frac{\mathbf{E}(\omega)}{\omega_{0}^{2}-\omega^{2}-j\omega\gamma}$ (S12)

The electric polarization is expressed as:

$\mathbf{P}=Nq\mathbf{r}\left(\omega\right)=\frac{Nq^{2}}{m}\frac{\mathbf{E}(\omega)}{\omega_{0}^{2}-\omega^{2}-j\omega\gamma}$ (S13)

Considering the electric susceptibility *χ*^*^_e_:

$\begin{matrix} \chi_{e}^{*}=\chi'_{e}+\chi''_{e}=\frac{\mathbf{P}}{\mathbf{E}\left(\omega\right)\varepsilon_{0}} \\ \text{ }=\frac{Nq^{2}}{m\varepsilon_{0}}\frac{1}{\omega_{0}^{2}-\omega^{2}-j\omega\gamma} \\ \text{ }=\frac{\omega_{p}^{2}\left(\omega_{0}^{2}-\omega^{2}\right)}{\left(\omega_{0}^{2}-\omega^{2}\right)^{2}+\omega^{2}\gamma^{2}}+j\frac{\omega_{p}^{2}\omega\gamma}{\left(\omega_{0}^{2}-\omega^{2}\right)^{2}+\omega^{2}\gamma^{2}} \end{matrix}$ (S14)

Where the *ω*_p_ is the plasma frequency. Thus, the permittivity ε_r_ can be calculated as[3,9]:

$\begin{matrix} \varepsilon_{r}^{*}=\varepsilon'_{r}\text{+}\varepsilon''_{\text{r}}=1+\chi_{e} \\ \text{ }=1+\frac{\omega_{pe}^{2}}{\omega_{e}^{2}-\omega^{2}-j\omega\gamma} \\ \text{ =}1+\frac{\omega_{pe}^{2}(\omega_{e}^{2}-\omega^{2})}{\left(\omega_{e}^{2}-\omega^{2}\right)^{2}+j\omega\gamma}+j\frac{\omega_{pe}^{2}\omega\gamma}{\left(\omega_{e}^{2}-\omega^{2}\right)^{2}+\omega^{2}\gamma^{2}} \end{matrix}$ (S15)

Considering that the metasurface has a dielectric substrate with permittivity *ε*_b_, thus the complex permittivity of the metasurface with electric resonance is:

$\varepsilon_{r}^{*}=\varepsilon_{\text{b}}\left(1+\frac{\omega_{pe}^{2}(\omega_{e}^{2}-\omega^{2})}{\left(\omega_{e}^{2}-\omega^{2}\right)^{2}+j\omega\gamma}+j\frac{\omega_{pe}^{2}\omega\gamma}{\left(\omega_{e}^{2}-\omega^{2}\right)^{2}+\omega^{2}\gamma^{2}}\right)$ (S16)

For a dielectric material with a complex permittivity, its refractive index must also be a complex form, denoted by:

$n^{*}=n+j\kappa=\pm\sqrt{\varepsilon_{\text{b}}}\sqrt{\varepsilon_{r}^{*}\mu_{r}^{*}}=\sqrt{\varepsilon_{\text{b}}}\sqrt{(1+\chi_{e}^{*})\left(1+\chi_{m}^{*}\right)}$ (S17)

Considering that only electrical resonance occurs in the material, the magnetic susceptibility is ignored.

$\begin{matrix} n^{*}=\sqrt{\varepsilon_{\text{b}}}\sqrt{(1+\chi_{e}^{*})\left(1+\chi_{m}^{*}\right)}\approx\pm\sqrt{\varepsilon_{\text{b}}}\sqrt{(1+\chi_{e}^{*})}\approx\sqrt{\varepsilon_{\text{b}}}\left(1+\frac{\chi'_{e}}{2}+j\frac{\chi''_{e}}{2}\right) \\ \text{ }=\sqrt{\varepsilon_{\text{b}}}\left(1+\frac{1}{2}\frac{\omega_{pe}^{2}\left(\omega_{0e}^{2}-\omega^{2}\right)}{\left(\omega_{0e}^{2}-\omega^{2}\right)^{2}+\omega^{2}\gamma_{e}^{2}}+j\frac{1}{2}\frac{\omega_{pe}^{2}\omega\gamma}{\left(\omega_{e}^{2}-\omega^{2}\right)^{2}+\omega^{2}\gamma_{e}^{2}}\right) \end{matrix}$ (S18)

Thus,

$\left\{\begin{matrix} n\left(\omega\right)=\sqrt{\varepsilon_{\text{b}}}\left(1+\frac{1}{2}\frac{\omega_{pe}^{2}\left(\omega_{0e}^{2}-\omega^{2}\right)}{\left(\omega_{0e}^{2}-\omega^{2}\right)^{2}+\omega^{2}\gamma_{e}^{2}}\right) \\ \kappa\left(\omega\right)=\sqrt{\varepsilon_{\text{b}}}\left(\frac{1}{2}\frac{\omega_{pe}^{2}\omega\gamma}{\left(\omega_{0e}^{2}-\omega^{2}\right)^{2}+\omega^{2}\gamma_{e}^{2}}\right) \end{matrix}\right.$ (S19)

We further deform the real part of the refractive index n(ω) as

$\begin{matrix} n\left(\omega\right)=\sqrt{\varepsilon_{b}}\left(1+\frac{1}{2}\frac{\omega_{pe}^{2}\left(\omega_{0e}^{2}-\omega^{2}\right)}{\left(\omega_{0e}^{2}-\omega^{2}\right)^{2}\text{+}\omega^{2}\gamma_{e}^{2}}\right) \\ \text{ =}\sqrt{\varepsilon_{b}}\left(1+\frac{\frac{\omega_{pe}^{2}}{2\left(\omega_{0e}^{2}-\omega^{2}\right)}}{1\text{+}\frac{\gamma_{e}^{2}}{\left(\omega_{0e}^{2}-\omega^{2}\right)^{2}}\omega^{2}}\right) \end{matrix}$ (S20)

By comparing with the formula, it can be found that the form of refractive index has Debye relaxation form.

$\left\{\begin{matrix} n\left(\omega\right)=n_{\infty}+\frac{n_{s}-n_{\infty}}{1\text{+}\tau^{2}\omega^{2}} \\ \text{ }\tau=\left|\frac{\gamma_{e}}{\omega_{0e}^{2}-\omega^{2}}\right| \\ \text{ }n_{s}=n_{\infty}\left(1+\frac{\omega_{pe}^{2}}{2\left(\omega_{0e}^{2}-\omega^{2}\right)}\right) \\ \text{ }n_{\infty}=\sqrt{\varepsilon_{b}}\text{ } \end{matrix}\right.$ (S21)

Where the τ is the relaxation time, *n*_s_ is the static refractive index in low frequency, *n*_∞_ is the static refractive index in high frequency. Thus, the propagation phase imparted from the metasurface as the effective medium has also the Debye relaxation process:

 (S22)

The *φ*_s_(ω) and *φ͚*(ω) are calculated as:

 (S23)

**2. 1st-order Debye-relaxation induced by magnetic resonance**

Magnetic resonance also has a Lorenz resonance form similar to electrical resonance, except that magnetic resonance changes the permeability, while electrical resonance changes the permittivity. The permeability is expressed as:

$\mu_{r}^{*}=\mu'_{r}+j\mu''_{r}=1+\frac{\omega_{pm}^{2}}{\omega_{0m}^{2}-\omega^{2}+j\gamma_{m}\omega}$ (S24)

Where *ω_pm_* is the plasma frequency of the magnetic resonance, *ω*_0m_ is the resonance frequency of the magnetic resonance, *γ*_m_ is the loss of the magnetic resonance. For a dielectric material with a complex permeability, its refractive index must also be a complex form, denoted by:

$n^{*}=n+j\kappa=\pm\sqrt{\varepsilon_{\text{b}}}\sqrt{\varepsilon_{r}^{*}\mu_{r}^{*}}=\sqrt{\varepsilon_{\text{b}}}\sqrt{(1+\chi_{e}^{*})\left(1+\chi_{m}^{*}\right)}$ (S25)

Considering that only magnetic resonance occurs in the material, the electric susceptibility is ignored.

$\begin{matrix} n^{*}=\sqrt{\varepsilon_{\text{b}}}\sqrt{(1+\chi_{e}^{*})\left(1+\chi_{m}^{*}\right)}\approx\pm\sqrt{\varepsilon_{\text{b}}}\sqrt{(1+\chi_{m}^{*})}\approx\sqrt{\varepsilon_{\text{b}}}\left(1+\frac{\chi'_{m}}{2}+j\frac{\chi''_{m}}{2}\right) \\ \text{ }=\sqrt{\varepsilon_{\text{b}}}\left(1+\frac{1}{2}\frac{\omega_{pm}^{2}\left(\omega_{0m}^{2}-\omega^{2}\right)}{\left(\omega_{0m}^{2}-\omega^{2}\right)^{2}+\omega^{2}\gamma_{m}^{2}}+j\frac{1}{2}\frac{\omega_{pm}^{2}\omega\gamma}{\left(\omega_{m}^{2}-\omega^{2}\right)^{2}+\omega^{2}\gamma_{m}^{2}}\right) \end{matrix}$ (S26)

Thus, the refractive index can be written as:

$\left\{\begin{matrix} n\left(\omega\right)=\sqrt{\varepsilon_{\text{b}}}\left(1+\frac{1}{2}\frac{\omega_{pm}^{2}\left(\omega_{0m}^{2}-\omega^{2}\right)}{\left(\omega_{0m}^{2}-\omega^{2}\right)^{2}+\omega^{2}\gamma_{m}^{2}}\right) \\ \kappa\left(\omega\right)=\sqrt{\varepsilon_{\text{b}}}\left(\frac{1}{2}\frac{\omega_{pm}^{2}\omega\gamma}{\left(\omega_{0m}^{2}-\omega^{2}\right)^{2}+\omega^{2}\gamma_{m}^{2}}\right) \end{matrix}\right.$ (S27)

The *n*(*ω*) can be transformed as:

$\begin{matrix} n\left(\omega\right)=\sqrt{\varepsilon_{b}}\left(1+\frac{1}{2}\frac{\omega_{pm}^{2}\left(\omega_{0m}^{2}-\omega^{2}\right)}{\left(\omega_{0m}^{2}-\omega^{2}\right)^{2}\text{+}\omega^{2}\gamma_{m}^{2}}\right) \\ \text{ =}\sqrt{\varepsilon_{b}}\left(1+\frac{\frac{\omega_{pm}^{2}}{2\left(\omega_{0m}^{2}-\omega^{2}\right)}}{1\text{+}\frac{\gamma_{m}^{2}}{\left(\omega_{0m}^{2}-\omega^{2}\right)^{2}}\omega^{2}}\right) \end{matrix}$ (S28)

It can be seen *n*(*ω*) has Debye relaxation form and is expressed as below:

$\left\{\begin{matrix} n\left(\omega\right)=n_{\infty}+\frac{n_{s}-n_{\infty}}{1\text{+}\tau^{2}\omega^{2}} \\ \text{ }\tau=\left|\frac{\gamma_{m}}{\omega_{0m}^{2}-\omega^{2}}\right| \\ \text{ }n_{s}=n_{\infty}\left(1+\frac{\omega_{pm}^{2}}{2\left(\omega_{0m}^{2}-\omega^{2}\right)}\right) \\ \text{ }n_{\infty}=\sqrt{\varepsilon_{b}}\text{ } \end{matrix}\right.$ (S29)

Thus, the propagation phase imparted from the metasurface as the effective medium has also the Debye relaxation process:

 (S30)

Considering whether in mechanical system or electromagnetic system, resonance is always accompanied with phase lag. It can be seen the relaxation time depends on the angular frequency and resonance damping. When the angular frequency of incident wave is close to the frequency of electric resonance, the relaxation time increases sharply. Moreover, the relaxation time increases with the increase of damping of electric resonance. This is also consistent with resonance characteristics.

**S3: Design principle of the meta-atom**

The phase response of the QEAS meta-atom with different α and *x* are plotted in Fig. S2. For the variation of *x*, the phase response is plotted in Fig. S2(a). It can be seen that, for *y* $\in$[0.9 mm,1.3 mm], the phase response in the lower frequency band is steeper, while that in higher frequency band is moderate. On the contrary, for *x* $\in$[1.3 mm,2.1 mm], the phase variation slope in higher frequency band is steeper, while that in lower frequency band is more gradual. Compared to *x* and *y*, the change of *α* has the greatest influence on the phase value, but the change of phase dispersion is relatively moderate. Since *L* has little influence on the phase response except for some specific parameter combinations, the variation curve of *L* on the phase response is not shown in the figure. Therefore, we have observed that the dispersion engineering can be achieved as a result of the comprehensive coordination of the aforementioned four parameters.


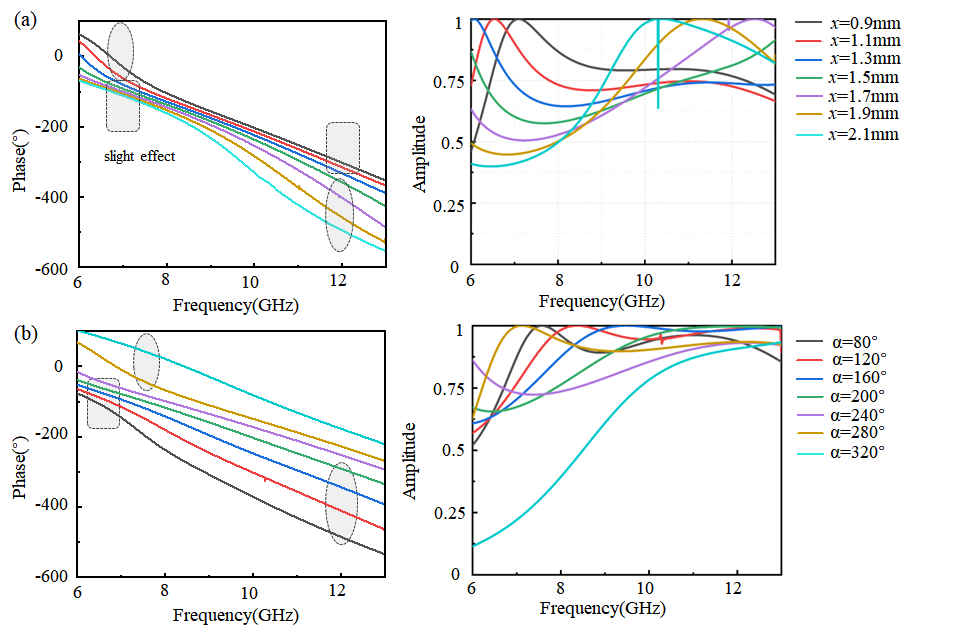


Fig. S2 the phase response to the different α and *x*: (a) The *x* variation, (b) the *α* variation. The left column is the phase response under RCP wave illumination, while the right column is the amplitude response under RCP wave illumination.

Table S1 The parameters of meta-atoms with constant phase gradient

| Meta-Atom Number | *L*(mm) | *α*(°) | *x*(mm) | *y*(mm) |
| --- | --- | --- | --- | --- |
| NO. Ⅰ-1 | 2.5 | 140.0 | 1.2 | 1.7 |
| NO. Ⅰ-2 | 1.9 | 140.0 | 1.9 | 1.8 |
| NO. Ⅰ-3 | 1.8 | 90.0 | 1.6 | 1.9 |
| NO. Ⅰ-4 | 2.5 | 92.0 | 1.9 | 2.2 |
| NO. Ⅰ-5 | 2.5 | 70.0 | 2.0 | 2.3 |
| NO. Ⅰ-6 | 2.5 | 290.0 | 2.0 | 1.5 |
| NO. Ⅰ-7 | 1.8 | 230.0 | 1.4 | 1.9 |
| NO. Ⅰ-8 | 1.8 | 200.0 | 1.2 | 2.1 |

Table S2 The parameters of meta-atoms with linearly increasing phase gradient

| Meta-Atom Number | *L*(mm) | *α*(°) | *x*(mm) | *y*(mm) |
| --- | --- | --- | --- | --- |
| NO. Ⅱ-1 | 2.5 | 183.0 | 1.5 | 2.4 |
| NO. Ⅱ-2 | 1.8 | 120.0 | 1.0 | 2.3 |
| NO. Ⅱ-3 | 1.7 | 110.0 | 1.7 | 2.2 |
| NO. Ⅱ-4 | 1.8 | 70.0 | 1.6 | 2.5 |
| NO. Ⅱ-5 | 2.5 | 320.0 | 1.0 | 1.9 |
| NO. Ⅱ-6 | 2.5 | 310.0 | 1.0 | 2.3 |
| NO. Ⅱ-7 | 1.8 | 210.0 | 1.0 | 1.7 |
| NO. Ⅱ-8 | 1.3 | 160.0 | 1.3 | 1.4 |
| NO. Ⅱ-9 | 1.1 | 140.0 | 1.1 | 1.6 |
| NO. Ⅱ-10 | 3.2 | 80.0 | 1.4 | 1.3 |
| NO. Ⅱ-11 | 2.5 | 85.0 | 1.9 | 1.2 |
| NO. Ⅱ-12 | 2.5 | 71.0 | 1.9 | 1.2 |


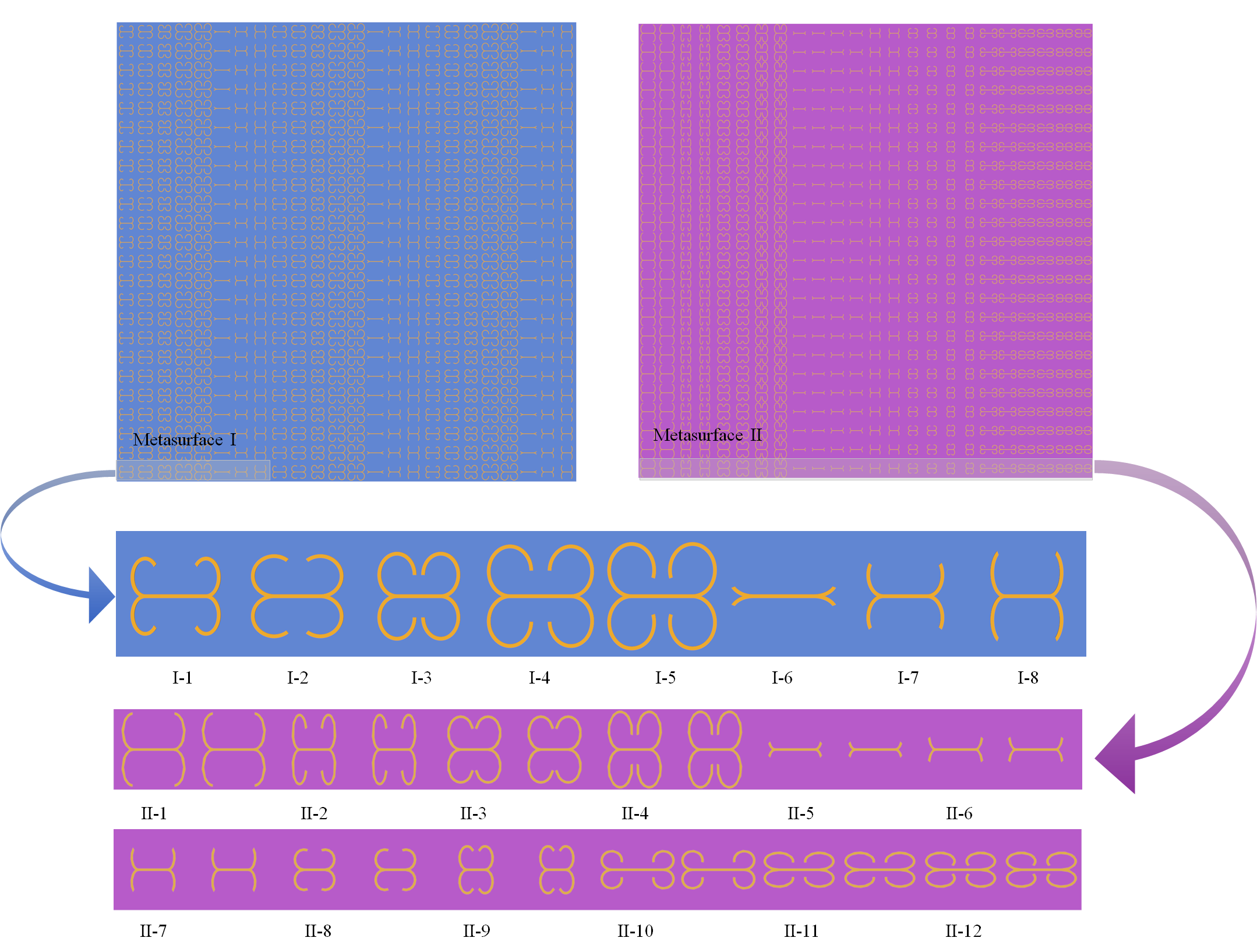


Fig. S3 The selected meta-atoms and the metasurfaces. The metasurface Ⅰ is with the desirable linear phase distribution while the the metasurface Ⅱ is with achromatic phase distribution.

**S4: Simulation result of linear and elliptical polarization focusing performance**

In general, an arbitrary polarization state *ζ* can be depicted in Poincar´e sphere and fully described by two parameters, namely, azimuth *ψ* and ellipticity *χ* of the polarization ellipse, respectively. Its Jones vector can be explicitly written in the form[11]:

$\xi\text{=}\left[\begin{matrix} \cos\psi\\ e^{i\chi}\sin\psi\end{matrix}\right]\text{ }\left\{\begin{matrix} \chi=0^{\circ},\text{ }\psi\text{=}0^{\circ}\text{ x polarization} \\ \chi=0^{\circ},\text{ }\psi\text{=9}0^{\circ}\text{ y polarization} \\ \chi=90^{\circ},\text{ }\psi\text{=}0^{\circ}\text{ RCP } \\ \chi=-90^{\circ},\text{ }\psi\text{=}0^{\circ}\text{ LCP} \\ \text{else }Elliptical\text{ }polarization \end{matrix}\right.$ (S31)

Different parameter values correspond to different polarization states and are already listed in the Eq. (S31). Considering that any linearly polarized wave with polarization angle *φ* can be decomposed into two orthogonal CP wave(LCP&RCP) components and the decomposition is expressed by the Jones vector:

$\begin{matrix} \xi\text{=}\left[\begin{matrix} \cos\psi\\ e^{i\chi}\sin\psi\end{matrix}\right]=\frac{\sqrt{2}}{2}\left(\cos\psi+ie^{i\chi}\sin\psi\right)\left(\frac{1}{\sqrt{2}}\left(\begin{matrix} 1 \\ -i \end{matrix}\right)\right)+\frac{\sqrt{2}}{2}\left(\cos\psi-ie^{i\chi}\sin\psi\right)\left(\frac{1}{\sqrt{2}}\left(\begin{matrix} 1 \\ i \end{matrix}\right)\right) \\ \text{ =}\frac{\sqrt{2}}{2}\left(\cos\psi+ie^{i\chi}\sin\psi\right)|L\rangle+\frac{\sqrt{2}}{2}\left(\cos\psi-ie^{i\chi}\sin\psi\right)|R\rangle\end{matrix}$ (S32)

where |L> and |R> are denoted LCP and RCP wave, respectively. Eq.(38) indicates that two orthogonal CP waves can be superposed to synthesis a wave with arbitrary polarization state ζ. Then, considering that the RCP and LCP components carry the phase *φ* with equal magnitude and the same sign. Then Eq.(S32) can be rewritten as:

 (S33)

It is indicated that once the LCP and RCP component carrying the same phase *φ,* the superposed wave ζ will also acquire the phase *φ*_g_. Therefore, the metasurface under linear- and elliptical- polarization wave illumination will also exhibit the same performance. To demonstrate it, the transverse electric field intensity distribution under LP and EP wave illumination are simulated and depicted in Fig. S4 and S5, respectively. The simulated results indicated that the two metasurface render chromatic and achromatic focus in the entire X band from 8.0 to 12.0 GHz, respectively, in good agree with the performance under CP wave illumination.


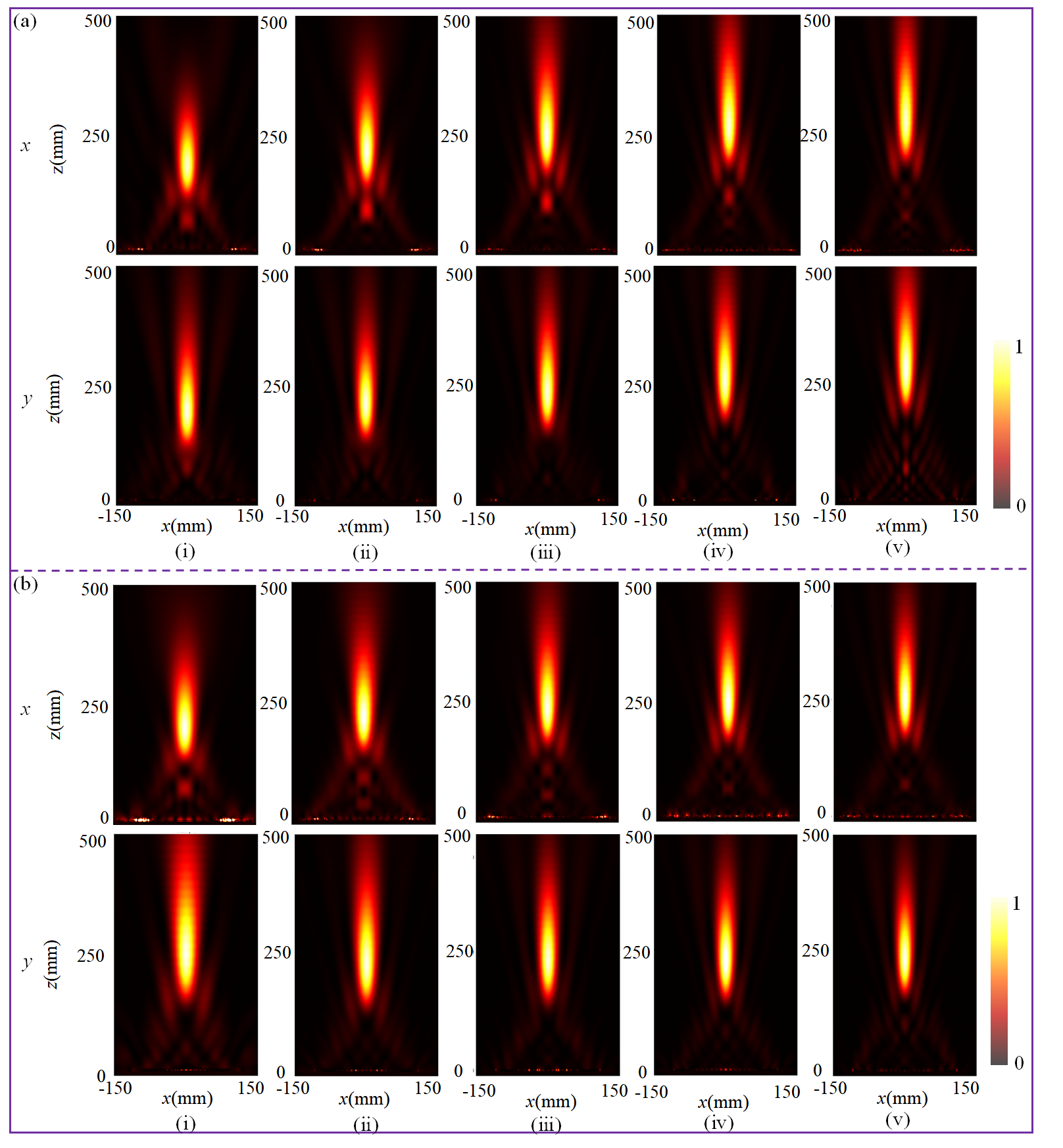


Fig. S4 The **|**E_||_**|**^2^ distribution under LP wave illumination at *y-o-z* plane. (a) chromatic metasurface(The top row is under x-polarization wave illumination, while the bottom row is under y-polarization wave)(b) achromatic metasurface(The top row is under x-polarization wave illumination, while the bottom row is under y-polarization wave). The column (ⅰ) 8.0 GHz, (ⅱ) 9.0 GHz, (ⅲ) 10.0 GHz, (ⅳ) 11.0 GHz, (ⅴ) 12.0 GHz.


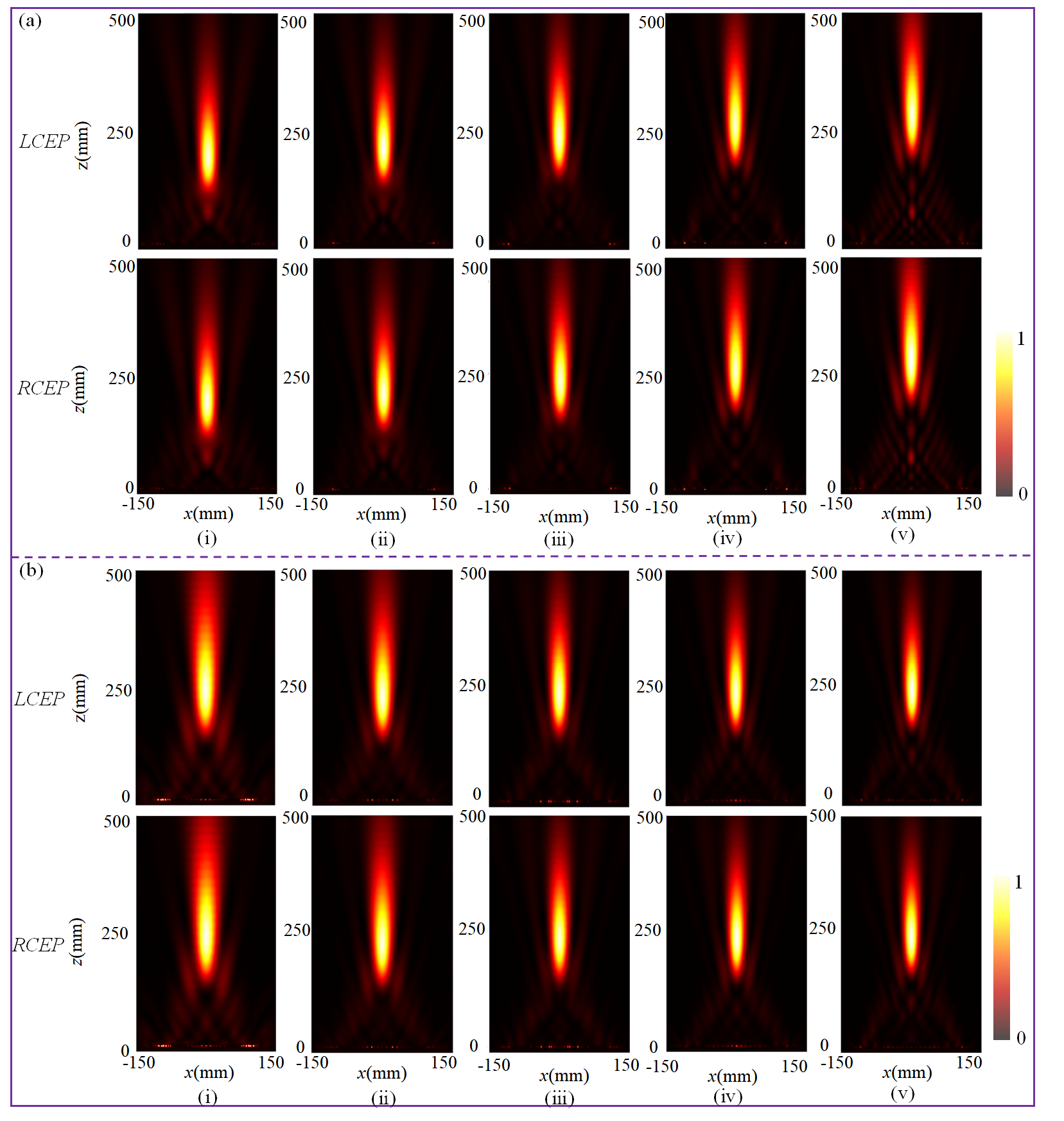


Fig. S5 The **|**E_||_**|**^2^ distribution under elliptical-polarization(EP) wave illumination at *y-o-z* plane(left-handed elliptical-polarization: LEP; right-handed elliptical-polarization: REP). (a) chromatic metasurface(The top row is under LEP wave illumination, while the bottom row is under REP wave)(b) achromatic metasurface(The top row is under LEP wave illumination, while the bottom row is under REP wave). The column (ⅰ) 8.0 GHz, (ⅱ) 9.0 GHz, (ⅲ) 10.0 GHz, (ⅳ) 11.0 GHz, (ⅴ) 12.0 GHz.

**Reference**

[1] A. K. Jonscher, “Dielectric relaxation in solids.” 1983.

[2] A. K. Jonscher, “universal relaxation law.” 1996.

[3] K. C. Kao. Dielectric phenomena in solids. Elsevier. 2004

[4] R. Liu, T. J. Cui, D. Huang, B. Zhao, D. R. Smith, Description and explanation of electromagnetic behaviors in artificial metamaterials based on effective medium theory. Physical Review E, 76(2), 026606 (2007).

[5] W. X. Jiang, Z. L. Mei, T. J. Cui. Effective medium theory of metamaterials and metasurfaces. Cambridge University Press, 2021.

[6] J. B. Pendry, A. J. Holden, W. J. Stewart, I. Youngs, Extremely low frequency plasmons in metallic mesostructures. Physical review letters, 76(25), 4773 (1996)

[7] J. B. Pendry, A. J. Holden, D. J. Robbins, W. J. Stewart, Low frequency plasmons in thin-wire structures. Journal of Physics: Condensed Matter, 10(22), 4785 (1998)

[8] D. R. Smith, J. B. Pendry, M. C. Wiltshire, Metamaterials and negative refractive index. Science, 305(5685), 788 (2004)

[9] D. C. Elton. The origin of the Debye relaxation in liquid water and fitting the high frequency excess response. Physical Chemistry Chemical Physics, 19(28), 18739 (2017)

[10] L. Zhu, Q. Wang, Novel ferroelectric polymers for high energy density and low loss dielectrics. Macromolecules, 45(7), 2937 (2012)

[11] Q. Fan, M. Liu, C. Zhang, W. Zhu, Y. Wang, P. Lin, F. Yan, L. Chen, H. J. Lezec, Y. Q Lu, A. Agrawal, T. Xu, Independent amplitude control of arbitrary orthogonal states of polarization via dielectric metasurfaces. Physical review letters, 125(26), 267402 (2020)
